# Alpha-synucleinopathy and neuropsychological symptoms in a population-based cohort of the elderly

**DOI:** 10.1186/s13195-015-0101-x

**Published:** 2015-04-13

**Authors:** Julia Zaccai, Carol Brayne, Fiona E Matthews, Paul G Ince, on behalf of the MRC Cognitive Function and Ageing Neuropathology Study

**Affiliations:** Institute of Public Health, University of Cambridge, Cambridge, UK; MRC Biostatistics Unit, Institute of Public Health, Cambridge, UK; Sheffield Institute for Translational Neuroscience, 385A Glossop Road, Sheffield, S10 2HQ UK

## Abstract

**Introduction:**

Studies with strong selection biases propose that alpha-synucleinopathy (AS) spreads upwards and downwards in the neuraxis from the medulla, that amygdala-dominant AS is strongly associated with Alzheimer’s disease (AD), and that a more severe involvement of the cerebral cortex is correlated with increasing risk of dementia. This study examines the association of AS patterns and observed neuropsychological symptoms in brains of a population-representative donor cohort.

**Methods:**

Brains donated in 2 out of 6 cognitive function and ageing study cohorts (Cambridgeshire and Nottingham) were examined. Over 80% were >80 years old at death. The respondents were evaluated prospectively in life for cognitive decline and dementia. Immunocytochemistry for tau and alpha-synuclein (using LB509 by Zymed Laboratories) was carried out in 208 brains to establish Braak stage and the pattern and severity of AS following the dementia with Lewy bodies (DLB) consensus recommendations. Dementia, specific neuropsychological measures as measured using the Cambridge cognitive examination, the presence of hallucinations and Parkinson’s disease were investigated.

**Results:**

Four patterns of AS were observed: no AS pathology (n = 92), AS pathology following the DLB consensus guidelines (n = 33, of which five were ‘neocortical’), amygdala-predominant AS (n = 18), and other AS patterns (n = 33). Each group was subdivided according to high/low neurofibrillary tangles (NFT) Braak stage. Results showed no association between dementia and these patterns of AS, adjusting for the presence of NFT or not. The risk of visual hallucinations shows a weak association with AS in the substantia nigra (odds ratio (OR) = 3.2; 95% confidence interval (CI) 0.5 to 15.5; *P* = 0.09) and amygdala (OR = 3.0; 95% CI 0.7 to 12.3; *P* = 0.07). The analysis is similar for auditory hallucinations in subcortical regions.

**Conclusions:**

Among the whole population of older people, AS does not increase the risks for dementia, irrespective of Braak stage of NFT pathology. There was no evidence that the pattern of AS pathology in cortical areas was relevant to the risk of hallucination. In general, the hypothesis that AS as measured using these methods *per se* is a key determinant of cognitive clinical phenotypes is not supported.

## Introduction

Diagnostic protocols for the neuropathological assessment of the ageing brain, for use in both research and clinical diagnosis, are based on the severity and anatomical distribution of lesions [1-3]. There is an expectation that ‘cases’ will be pathologically distinguishable from unimpaired individuals although the pathologies of Alzheimer’s disease (AD), Parkinson’s disease (PD) and Dementia with Lewy bodies (DLB) are routinely present in normal elderly brain donors [4-8]. Comparison of brain pathology in AD cases with DLB and PD cases suggests that Lewy body (LB) pathology contributes significantly to dementia [9-13]. However, there is no evidence-based consensus for a threshold of AS that will distinguish demented from non-demented people [5,14,15]. The prevalence of α-synucleinopathy (AS), the substrate of LB pathology, is reported to vary between 2% to 12% in people without dementia at death and from 12% to 61% in people with dementia [5,14,16-18]. Limited AS in cortical and subcortical areas is variably interpreted as presymptomatic disease [7,19] or non-specific age-related degeneration.

This previous work on the clinicopathological association of alpha-synucleinopathy is based on cohorts derived from secondary referral, memory clinics or community volunteers. These methods of selection introduce underestimated biases that prevent extrapolation of the conclusions to the whole population of elderly people. In general the impact of pathology is exaggerated in such studies [20]. In this study the association between AS, AD pathology, dementia, clinical PD and neuropsychological symptoms was assessed in a truly population-representative sample free of selection biases associated with clinical diagnosis, symptoms or access to health and social care facilities.

## Methods

### Medical Research Council Cognitive Function and Ageing Study

The Medical Research Council Cognitive Function and Ageing Study (MRC CFAS) has been fully described elsewhere [21]. It is a longitudinal, prospective, population-based cohort study initiated in 1989. At each of five centres in England and Wales random samples of about 2,500 people, 64-years old and older (with an 82% response rate), agreed to a structured initial interview. Structured interviews collected details of sociodemographic status, general health, Mini Mental State Examination (MMSE) [22] and functional ability. Approximately 20% of the sample completed a more detailed diagnostic assessment, including full mood and organicity sections of the Geriatric Mental State (GMS) Automated Geriatric Examination for Computer Assisted Taxonomy (AGECAT) [23] and the Cambridge Cognitive Examination (CAMCOG) [24]. Further waves of interviewing (both screening and assessment) were conducted at two, five and nine years after baseline on the survivors. In a sixth centre 5,200 people, 65-years old and older, were interviewed using the same assessment interviews with follow-up at one to two, three to four, five to six, and nine years later. The CFAS Neuropathology Study is based on a brain donor programme established in 1992. Individuals selected for the assessment interviews were approached by a trained liaison officer for brain donation. The donation programme was discussed with the respondent and their family or carer as appropriate. All participants in the study gave their informed consent. This analysis is based on the burden and anatomical distribution of AS in all donations from two MRC CFAS centres before July 2003 (Nottingham and Cambridgeshire, n = 208). Research was carried out after approval from the Eastern Multi Centre Research Ethics Committee based at Papworth Hospitals NHS Trust (reference 03/5/017).

### Postmortem procedure and anatomical sampling

Pathological evaluation of the Neuropathology Study cohort had previously been carried out using the Consortium to Establish a Registry for Alzheimer’s Disease (CERAD) protocol [1] and each brain was assigned a Braak score for neurofibrillary tangles [2]. At autopsy samples of brain tissue were removed for frozen storage. The remainder of the brain was fixed for standardised assessment using paraffin-embedded tissues. For the evaluation of synucleinopathy a hierarchical sampling strategy was adopted based on evaluation of the midbrain (substantia nigra), medulla (dorsal efferent nucleus of the vagus nerve, medullary reticular formation) and amygdala to detect AS pathology. This method of screening was selected because literature at the time of the study indicated that no cases had been reported in which AS was present in any brain region but absent from these three screening areas [3,25-29]. If at least one positive α-synuclein immunoreactive profile was found in a screening area five additional areas were investigated (cingulate gyrus, entorhinal cortex and hippocampus, frontal cortex, parietal cortex and temporal cortex), comprising the DLB Consensus recommendations for the evaluation of AS [30].

### Immunocytochemistry for alpha-synuclein, quantification

Microwave antigen retrieval (citric acid 10 mM pH6) of 6 μm brain sections was followed by immunocytochemistry using a monoclonal antibody to mouse alpha-synuclein (LB509, Zymed Laboratories Inc; San Francisco, CA USA; dilution 1/200; incubated at 4°C overnight). Incubation with biotinylated secondary antibody (1/250) for 1.5 hours at room temperature was followed by ABC Elite Mouse IgG reagents (Vector Laboratories, Burlingame, CA, USA) for one hour. Reaction product was visualised using Vector Nova Red (Vector Laboratories) according to the manufacturer’s protocol. All sections were counterstained with haematoxylin and mounted in DPX. Interpretation of the appearances was jointly established with propidium iodide (PI). Acceptable inter- and intra-rater reliability was previously reported in this study [31]. AS pathology was then rated by a single investigator (JZ). The microscopic field with the highest density of AS was identified and the number of AS profiles was counted at x200 magnification. Spherical and densely stained structures were considered to be LB irrespective of intra- or extracellular localisation. Elongated AS-positive profiles were considered to be Lewy neurites (LN). Two consecutive sections from each block were assessed and the highest density score retained for the statistical analysis. In the final analysis, results were dichotomised to AS present versus absent.

Each brain was allocated to a pathological category as shown in Table 1. These categories were based on the presence or absence of neurofibrillary tangles (NFT) and AS. NFT pathology was dichotomised on the basis of Braak stage (Low - stages I-III versus High - stages IV-VI). AS was divided into three categories based on previous work in this cohort:

1. Cases that conform to the hierarchy of anatomical spread of AS implicit in the DLB Consensus protocol for the evaluation of AS [30], which also implies that these cases conform to the Braak staging hypothesis proposed for AS [3]. Some analyses are based on the stratification of this group into those with DLB ‘limbic’ and ‘neocortical’ disease and those with AS confined to midbrain/brainstem structures;
2. Cases that conform to ‘amygdala-predominant’ AS implying that amygdala AS is prominent and that involvement of neocortical, brainstem and midbrain structures is limited and patchy [31];
3. Cases with an atypical AS distribution, predominantly those with mild to severe cortical involvement in the absence of AS in one of the lower hierarchic anatomical sites (for example, substantia nigra, locus ceruleus, dorsal vagus nucleus). For example, this category includes cases with prominent AS involvement observed in the substantia nigra and frontal cortex but not in other investigated areas, or cases with prominent AS involvement observed in the vagus nucleus and parietal cortex only. These also include six cases corresponding to the ‘cerebral-form of DLB’ [32] where AS was observed in the neocortex predominantly.

**Table 1:** **Pathology subgroups based on α-synucleinopathy (AS) and severity of neurofibrillary tangle (NFT) pathology**

**Neuropathology group**		**Criteria**	**Number**
No pathology		No AS; Braak stages I-III NFT	49
NFT only		No AS; Braak stages IV-VI NFT	43
AS		At least 1 LB or LN in any region	84
AS	+ high NFT	AS + Braak stages IV-VI NFT	32
AS	+ low NFT	AS + Braak stages I-III NFT	52
AS DLB	+ high NFT	AS pattern consistent with DLB Consensus; Braak stages IV-VI NFT	13
AS DLB	+ low NFT	AS pattern consistent with DLB Consensus; Braak stages I-III NFT	20
AS Amygdala-predominant	+ high NFT	amygdala-predominant AS pattern: Braak stages IV-VI NFT	9
AS Amygdala-predominant	+ low NFT	amygdala-predominant AS pattern: Braak stages I-III NFT	9
AS Other pattern	+ high NFT	AS pattern not consistent with DLB consensus or amygdala-predominant AS; Braak stages IV-VI NFT	10
AS Other pattern	+ low NFT	AS pattern not consistent with DLB consensus or amygdala-predominant AS: Braak stages I-III NFT	23

DLB, dementia with Lewy bodies.

Previous findings in this cohort showed that there was no association between these different AS staining patterns and Braak stage for NFT [31]. Thirty-two brains were excluded from the final analysis due to missing pathological or clinical data.

### Dementia and other outcomes

Dementia was defined using a combination of the AGECAT algorithm, death certificates and informant interviews, including retrospective informant interviews after death (RINI). Ten percent of participants were classified as having unknown dementia status on the basis that both the time between the last interview and death was too long to exclude incident dementia (over six months) and that data collected during informant interviews were inconclusive. The presence or absence of hallucinations (visual and auditory) or PD was based on self-reported symptoms and informant interview data.

### Specific neuropsychological measures

General cognitive function was assessed using the MMSE. A score of 21 or less was used to indicate the likely presence of dementia. The CAMCOG neuropsychological battery includes seven areas of cognition: orientation, language (comprehension and expression), memory (learning, recent and remote), attention/calculation, praxis, abstract thinking and perception. Cut-off scores for each domain subscales were taken as values falling below the 25th percentile cut-point in the whole of the non-demented MRC CFAS population. Results are presented for a total CAMCOG score (all seven areas) and for CAMCOG areas of attention/calculation, language (comprehension and expression), abstract thinking and perception. CAMCOG cut-off points were 83 out of a maximum score of 103, 5 out of 8 (attention/calculation), 25 out of 30 (language), 5 out of 8 (abstract thinking) and 6 out of 8 (perception). Informant and interview data were analysed for the presence or absence of visual hallucination, auditory hallucination and a self-reported diagnosis of Parkinson’s disease.

### Statistical analysis

MRC CFAS has released data at defined points. For this study, version 8.2 of the core MRC CFAS dataset and version 3.3 of the neuropathology dataset were used. All statistical work was conducted in Stata Software version 9.0 (Stata Corporation, College Station, TX, USA). Odds ratios and Fischer’s exact (chi squared) tests were applied to estimate the strength of any association between two observations. High *P*-values are associated with a higher likelihood that there is no significant difference between compared groups. All *P*-values are two sided.

## Results

### Demographics and AS staging patterns of the study cohort

The sample used in this analysis is representative of all MRC CFAS centres (Table 2). It includes a wide range of age-related neuropathology and a spectrum of clinical states including dementia and normal cognition. The sample contains a high proportion of the ‘oldest-old’ in whom the burden of dementia and neurodegeneration is highest.

**Table 2:** **Demographics of the respondents**

**Demographic characteristic**	**All MRC CFAS (Number = 451)**		**Cambridgeshire and Nottingham cohorts (Number = 208)**		**NFT only (Number = 43)**		**AS + NFT (Number = 32)**		**AS only (Number= 52)**	
Sex										
Men	188	(41)	123	(59)	10	(23)	16	(50)	21	(40)
Women	268	(59)	85	(41)	33	(74)	16	(50)	31	(60)
Age at death										
70 to 79	89	(19)	40	(19)	5	(12)	4	(12)	15	(29)
80 to 89	204	(49)	102	(49)	19	(44)	16	(50)	28	(54)
90 to 105	163	(32)	66	(32)	19	(44)	12	(48)	9	(17)
Education										
<10 years	318	(69)	159	(76)	33	(77)	26	(81)	35	(67)
10+ years	138	(31)	49	(24)	10	(23)	6	(19)	17	(33)
Social class										
Non manual	165	(36)	79	(38)	17	(39)	12	(37)	17	(33)
Manual	234	(51)	110	(33)	21	(49)	16	(50)	30	(58)
Unclassified/Army	57	(13)	19	(9)	5	(12)	4	(13)	5	(9)
Dementia status^a^										
Dementia	242	(53)	105	(50)	30	(70)	24	(75)	18	(34)
No dementia	184	(40)	83	(40)	12	(28)	6	(19)	27	(53)
Unknown^b^	30	(7)	20	(10)	1	(2)	2	(6)	7	(13)

^a^Determined using AGECAT organicity scores, informants interview items and death certificates; ^b^unknown dementia status was assigned on the basis that both the time between the last interview and death was too long to exclude incident dementia (over six months) and the data collected from interviews were inconclusive. AS, alpha-synucleinopathy; MRC CFAS, Medical Research Council Cognitive Function and Ageing Study; NFT, neurofibrillary tangles.

Among the 176 brain donors included in this analysis the four patterns of AS were distributed as follows: (1) no AS pathology (n = 92); (2) AS pathology following the DLB Consensus guidelines (n = 33; of which 5 were ‘neocortical’); (3) amygdala-predominant AS (n = 18) [28]; (4) other AS patterns (n = 33). Each group was subdivided according to the presence of NFT as defined by NFT Braak stage as dichotomised to low or high score (Table 1).

### AS and dementia or features of DLB

LB pathology was found in 34% of those without dementia during life and in 45% of those with dementia. The odds ratio for risk of dementia in association with LB is 1.6 (95% confidence interval 0.8 to 3.0, *P* = 0.1). There is a significant association between self-reported Parkinson’s disease and the presence of AS in substantia nigra, amygdala and the parietal neocortex (Table 3). There is also weak evidence of involvement of the cingulate cortex. These findings are consistent with the expected associations from the literature. The risk of visual hallucination in this population cohort shows weak evidence of an association with AS in substantia nigra (OR = 3.2; 95% CI 0.5 to 15.5; *P* = 0.09) and amygdala (OR = 3.0; 95% CI 0.7 to 12.3; *P* = 0.07). The analysis is similar for auditory hallucinations in subcortical regions. The odds ratios for visual hallucinations are higher in cortical regions compared to those for auditory hallucination but the small sample size restricts the statistical power of this analysis.

**Table 3:** **Relationship between α-synucleinopathy (AS) and a diagnosis of Parkinson’s disease (PD), visual hallucinations (VH) and auditory hallucinations (AH)**

**Brain area**	**% (brains showing AS)**											
	**No PD (n = 201)**	**PD (n = 7)**	**OR (95% CI)**	***P***	**No VH (n = 196)**	**VH (n = 12)**	**OR (95% CI)**	***P***	**No AH (n = 194)**	**AH (n = 14)**	**OR (95% CI)**	***P***
Substantia nigra	11	50	7.8 (1.0 to 60.6)	<0.01	12	30	3.2 (0.5 to 15.5)	0.09	11	31	3.5 (0.7 to 13.9)	0.04
Vagus nucleus	21	43	2.9 (0.4 to 17.8)	>0.2	21	25	1.2 (0.2 to 5.3)	>0.2	22	36	2.2 (0.5 to 7.8)	>0.2
Amygdala	22	57	4.7 (0.7 to 33.4)	0.03	22	45	3.0 (0.7 to 12.3)	0.07	22	36	1.9 (0.5 to 6.9)	>0.2
Frontal cortex	2	0			2	0			2	0		
Temporal cortex	2	0			2	8	4.0 (0.1 to 44.2)	>0.2	2	0		
Parietal cortex	3	29	11.1 (0.9 to 82.3)	<0.01	4	8	2.1 (0.04 to 18.5)	>0.2	4	7	1.8 (0.04 to 15.2)	>0.2
Hippocampus	2	0			2	0			2	0		
Entorhinal cortex	6	0	2.1 (0.04 to 18.2)	>0.2	7	0			6	7	1.2 (0.02 to 9.1)	>0.2
Cingulate cortex	3	14	5.4 (0.1 to 57.2)	>0.2	3	8	2.9 (0.05 to 27.1)	>0.2	3	7	2.4 (0.05 to 22.2)	>0.2

CI, confidence interval; OR, odds ratio.

### Association between AS staging pattern and neuropsychological symptoms

An initial analysis (not shown) compared the association of cognitive outcome variables between cases with neocortical and limbic patterns of AS according to the DLB Consensus guidelines and those with only brainstem involvement. In this population-based sample, none of the outcomes were significantly different for these two groups and they were combined in the analysis as the ‘DLB Consensus pattern’ group. Odds ratios for the association between neuropsychological symptoms and the different pathology groups are presented in Table 4. Adjusting for age, education, the presence of any vascular pathology, or a CAMCOG assessment performed more than two years before death, did not change the results. There was no association between AS and dementia (OR = 0.88; 95% CI 0.35 to 2.22) or between AS and low MMSE in cases with low NFT Braak stage (OR = 0.96; 95% CI 0.41 to 2.26). There was no additional risk of cognitive impairment when comparing brains with combined AS and NFT (OR for dementia = 4.63; 95% CI 1.52 to 15.1) to brains with NFT only (OR = 3.68; 95% CI 1.37 to 10.08). Among the three AS pathology groups with low NFT Braak stage, the group conforming to DLB Consensus guidelines was more likely to be demented (OR = 3.24; 95% CI 0.87 to 13.47). Brains classified as ‘neocortical’ DLB by the DLB Consensus guidelines, irrespective of NFT (n = 5), included only two with dementia. None of the nine brains with amygdala-predominant AS in the absence of NFT had dementia. These sub-groups are too small for statistical analysis. The cases with AS only in neocortical areas (‘cerebral form of DLB’; n = 6) [32], irrespective of NFT Braak stage, had a high likelihood of being demented (five of six), and all had low MMSE scores. However, there was no evidence of an association between the presence of this ‘cerebral-form of DLB’ and individual domains of CAMCOG scores. Braak stage in the five demented cases was: 0 (one case); 3 (one case); 5 (two cases); 6 (one case).

**Table 4:** **Association between neuropsychological symptoms and pathology groups (when compared to associations with a no pathology group, unadjusted)**

**Neuropsychological measure**	**High NFT only**				**AS + high NFT**				**AS + low NFT**		
	**n**	**N**	**OR 95% CI**	***P***	**n**	**N**	**OR 95% CI**	***P***	**n**	**N**	**OR 95% CI**
MMSE score <21	32	43	3.53 (1.30 - 10.01)	<0.01	23	32	2.21 (0.81 - 6.27)	>0.2	25	52	0.96 (0.41 - 2.26)
Dementia	30	43	3.68 (1.37 - 10.08)	<0.01	24	32	4.63 (1.52 - 15.07)	<0.01	17	52	0.88 (0.35 - 2.22)
Low CAMCOG Attention	25	43	1.29 (0.41 - 4.27)	>0.2	15	32	0.69 (0.21 - 2.32)	>0.2	23	52	0.56 (0.20 - 1.54)
Low CAMCOG Language^a^	27	43	2.05 (0.56 - 8.46)	>0.2	21	32	1.99 (0.49 - 9.68)	>0.2	26	52	0.7 (0.24 - 2.02)
Low CAMCOG Abstract thinking	18	43	1.93 (0.68 - 5.51)	>0.2	13	32	1.62 (0.53 - 5.02)	>0.2	20	52	1.5 (0.57 - 4.00)
Low CAMCOG Perception	27	43	2.33 (0.70 - 8.47)	>0.2	16	32	0.83 (0.27 - 2.63)	>0.2	31	52	2.01 (0.66 - 6.39)
Low CAMCOG Total^b^	29	43	3.67 (0.83 - 22.20)	>0.2	19	32	1.44 (0.38 - 6.13)	>0.2	26	52	0.82 (0.28 - 2.43)
	**DLB Consensus + high NFT**				**Amygdala-predominant + high NFT**				**other AS pattern + high NFT**		
MMSE score <21	10	13	3.21 (0.70 - 19.89)	>0.2	7	9	2.24 (0.44 - 14.76)	>0.2	6	10	1.44 (0.30 - 7.77)
Dementia	10	13	4.5 (0.96 - 28.04)	0.06	7	9	4.72 (0.77 - 49.99)	0.07	7	10	4.72 (0.77 - 49.99)
Low CAMCOG Attention	7	13	1.45 (0.23 - 16.15)	>0.2	4	9	0.41 (0.07 - 2.66)	>0.2	4	10	0.55 (0.08 - 4.40)
Low CAMCOG Language^a^	9	13	3.41 (0.38 -163.00)	>0.2	7	9	2.66 (0.28-130.36)	>0.2	5	10	0.95 (0.13 - 11.36)
Low CAMCOG Abstract thinking	4	13	1.2 (0.20 - 6.54)	>0.2	5	9	1.88 (0.34 - 10.87)	>0.2	4	10	2 (0.29 - 15.30)
Low CAMCOG Perception	8	13	2.07 (0.34 - 22.35)	>0.2	5	9	0.65 (0.12 - 3.85)	>0.2	3	10	0.39 (0.05 - 2.71)
Low CAMCOG Total^b^	8	13	3.03 (0.33 -146.68)	>0.2	5	9	0.63 (0.10 - 4.81)	>0.2	6	10	2.28 (0.23-114.05)
	**DLB Consensus + low NFT**				**Amygdala-predominant + low NFT**				**other AS pattern + low NFT**		
MMSE score <21	13	20	1.39 (0.45 - 4.38)	>0.2	1	9	0.12 (0.00 - 1.04 )	>0.2	11	23	1.32 (0.40 - 4.46)
Dementia	12	20	3.24 (0.87 - 13.47)	0.09	0	9	0 (0.00 - 0.62)	>0.2	5	23	0.56 (0.13 - 2.09)
Low CAMCOG Attention	10	20	0.83 (0.20 - 3.78)	>0.2	1	9	0.07 (0.00 - 0.70)	>0.2	12	23	0.83 (0.22 - 3.35)
Low CAMCOG Language^a^	9	20	0.57 (0.14 - 2.45)	>0.2	5	9	0.95 (0.13 - 11.36)	>0.2	12	23	0.76 (0.20 - 3.11)
Low CAMCOG Abstract thinking	6	20	1 (0.24 - 3.91)	>0.2	2	9	0.6 (0.05 - 4.29)	>0.2	12	23	3 (0.82 -11.68)
Low CAMCOG Perception	9	20	0.93 (0.23 - 4.26)	>0.2	6	9	3.11 (0.32-153.14)	>0.2	16	23	4.15 (0.77 - 41.44)
Low CAMCOG Total^b^	7	20	0.44 (0.10 - 2.01)	>0.2	6	9	2.28 (0.23-114.05)	>0.2	13	23	0.99 (0.25 - 4.38)

^a^Comprehension and Expression; ^b^over seven CAMCOG domains. AS, alpha-synucleinopathy; CAMCOG, Cambridge Cognitive Examination; CI, confidence interval; DLB, dementia with Lewy bodies; MMSE, Mini Mental State Examination; NFT, neurofibrillary tangles; OR, odds ratio.

Analysis of the risk associated with sub-threshold performance in individual domains of CAMCOG were not shown to be elevated above the control ‘no pathology’ group even in cases with high NFT Braak stage or combined AS and high NFT Braak stage. This reflects the sample sizes and the likely lack of power to detect an effect. It is interesting that in the AS cases with low NFT Braak stage the risk of subthreshold performance in the perceptual domain was increased for those with amygdala predominant disease (OR = 3.11; 95% CI 0.32 to 153.1) and the atypical AS group (OR = 4.15; 95% CI 0.77 to 41.44) but the robustness of this finding needs to be confirmed in a larger study.

## Discussion

We report the clinicopathological correlation of AS in a cohort of brains from older people donated from a population-based study. In this cohort of CFAS respondents, a reliable diagnosis of cognitive state at death was possible in 90%: 40% were non-demented at death and 50% demented. AS was present in 39% of the 208 brains examined. We have previously shown that AS in the population does not always conform to the DLB Consensus model of hierarchic spread, or the similar model implicit in proposed Braak staging of AS [3,30,31]. Only half of the brains with AS in this sample conform to DLB Consensus or Braak staging. A further 29% conform to the more recent concept of amygdala-predominant AS [33,34]. However, one in five old people with AS show a pattern that is not typical of either of these models, including approximately 3% who have AS largely restricted to the neocortex.

Previous studies reporting on a relationship between AS and neurologic and neuropsychological outcomes generally make a number of assumptions. These include a systematic hierarchical spread of disease upwards and downwards in the neuraxis from the medulla, a strong association between amygdala-predominant AS and Alzheimer’s disease, and the expectation that more severe involvement of the cerebral cortex is correlated with increasing risk of dementia [2,25-29,31,33-37]. There is also emerging evidence that AS is present in aged brains without neuro-psychiatric disorders or extra-pyramidal symptoms [6,7,38]. These variations can be explained by the fact that the research studies that underpin these assumptions did not involve the assessment of true population-based cohorts so that they emerged from, and have been tested in, cohorts influenced by selection biases associated with clinical referral, clinical case selection, hospitalisation or volunteer recruitment strategies, and in addition, to selection related to the pathological findings. The possibility therefore arises that inter-individual variation in pathophysiological responses might account for the differences seen between disease-based and population-based observations. It is thus possible that individuals who develop the clinicopathological features of a Lewy Body disorder (for example, PD and DLB) are predisposed to enable hierarchic spread of synucleinopathy in the brain whereas those chiefly encountered in population-based samples, and large unselected autopsy series, with atypical distribution of pathology and absent clinical features somehow resist such propagation.

In the present analysis we show that, among the whole population of elderly people, AS does not increase the overall risk for dementia, nor low MMSE or low CAMCOG scores, irrespective of whether it is associated with high or low Braak stage of NFT pathology. In AS cases with a low NFT Braak stage, the group showing pathological spread of AS consistent with the anatomical hierarchy implicit in the DLB Consensus and Braak staging, had an increased risk of dementia (OR = 3.24; 95% CI 0.87 to 13.47) compared to the amygdala-predominant (OR = 0; 95% CI 0.0 to 0.62) and atypical AS groups (OR = 0.56; 95% CI 0.13 to 2.09). This observation may in part explain the much more robust correlations with cognitive status reported in many cohort studies that largely concentrate on this subgroup of synucleinopathy. In addition, the cases with the so-called ‘neocortical-form of DLB’ were very likely to be demented (five of six), although three of these five cases had a high NFT Braak stage. Nonetheless, the overall risk of dementia in AS was not significantly associated with cortical compared to subcortical pathology. There was no relationship between any of the domains of cognition tested within the CAMCOG battery and AS in specific brain regions either with high or low NFT Braak stages. There was an expected relationship between self-reported PD and AS in the substantia nigra although self-reporting of PD in MRC CFAS, albeit suitable as a screening tool, was also found to identify a heterogeneous group of movement disorder patients [39]. This relationship also extended to the amygdala and parietal cortex with some evidence in the cingulate cortex. This latter association needs to be tested again with greater power or by using a different approach. These data reflect the probable greater concordance of the Braak staging model in the group with a clinicopathological diagnosis of Lewy body PD since the hypothesis was developed through the study of that disease together with two cohorts derived from secondary referrals selected on the basis of either AS pathology in the absence of known clinical disorder or absence of AS pathology in the brainstem with no clinical record of neurological disease [3]. Finally, there was evidence for an excess of visual hallucination in association with AS compared to auditory hallucination but both were associated with substantia nigra involvement. There was no evidence that the pattern of AS pathology was relevant to the risk of hallucination and no relationship with cortical pathology.

Although this is a relatively large cohort, the population sampling frame means that the power of the study is more limited in assessing the relationship between AS and specific neuropsychological features because the sample is not enriched either for cases with more extensive AS involving cortical regions or for specific clinical features such as hallucination. Another point that limits the conclusions of this study is that AS may have been underestimated in this group as its pathology in the olfactory bulb was not included in the initial screening process [40]. There is also an inherent underestimation of AS as a result of the study design in that solely two consecutive sections from each area were included in the assessment process. However, cortical pathology is present, and for most clinical correlates equally prevalent, in cases with and without these specific features.

## Conclusions

In general the hypothesis that AS *per se* is a key determinant of cognitive clinical phenotypes is not supported in this rare study of clinicopathological correlations of AS in a cohort of brains from older people donated from a population-based study.

## Abbreviations

AD: Alzheimer’s disease
AGECAT: automated examination for computer assisted taxonomy
AS: alpha-synucleinopathy
CAMCOG: Cambridge cognitive examination
CERAD: Consortium to Establish a Registry for Alzheimer’s Disease
CI: confidence interval
DLB: Dementia with Lewy bodies
GMS: Geriatric Mental State
LB: Lewy bodies
MMSE: Mini Mental State Examination
MRC CFAS: Medical Research Council Cognitive Function and Ageing Study
NFT: neurofibrillary tangles
OR: odds ratio
PD: Parkinson’s disease
RINI: Retrospective informant interviews after death
